# Exploiting Microbeams for Membrane Protein Structure Determination

**DOI:** 10.1007/978-3-319-35072-1_8

**Published:** 2016-04-28

**Authors:** Anna J. Warren, Danny Axford, Neil G. Paterson, Robin L. Owen

**Affiliations:** Diamond Light Source, Harwell Science and Innovation Campus, Didcot, OX11 0DE UK

**Keywords:** Microfocus macromolecular crystallography, Membrane proteins, *In situ* data collection, Instrumentation, X-ray microtomography

## Abstract

A reproducible, and sample independent means of predictably obtaining large, well-ordered crystals has proven elusive in macromolecular crystallography. In the structure determination pipeline, crystallisation often proves to be a rate-limiting step, and the process of obtaining even small or badly ordered crystals can prove time-consuming and laborious. This is particularly true in the field of membrane protein crystallography and this is reflected in the limited number of unique membrane protein structures deposited in the protein data bank (less than 650 by June 2016 – http://blanco.biomol.uci.edu/mpstruc). Over recent years the requirement for, and time and cost associated with obtaining, large crystals has been partially alleviated through the development of beamline instrumentation allowing data collection, and structure solution, from ever-smaller crystals. Advances in several areas have led to a step change in what might be considered achievable during a synchrotron trip over the last decade. This chapter will briefly review the current status of the field, the tools available to ease data collection and processing, and give some examples of exploitation of these for membrane protein microfocus macromolecular crystallography.

## Recent Developments in Instrumentation and Data Collection

Beamlines at third generation synchrotrons provide small and highly intense X-ray beams. This has been made possible due to developments in both machine and beamline instrumentation. On the machine side, a decrease in the emittance of the electron beam to typically less than 5 nm rad in the horizontal and less than 0.1 nm rad in the vertical, represents a reduction of more than an order of magnitude when compared to second generation sources. Continuous top-up, pioneered at the Swiss Light Source (Ludeke and Munoz 2002), provides a steady X-ray flux over the duration of an experimental visit and provides a constant power load on beamline components and aids thermal stability.

The provision of low divergence, high brilliance X-ray beams means a number of approaches can be used to achieve a microbeam. A high flux density source means that apertures can be used to reduce the beamsize at the sample to ~10 microns without the remaining flux dropping to zero. Alternatively, focusing elements can be used to achieve a microbeam containing all, or nearly all, of the full flux provided by the source. The different approaches for achieving microbeams are summarised further in (Evans et al. 2011b; Smith et al. 2012), but rapid developments in areas such as mirror fabrication mean that even beamlines nominally not dedicated to microfocus macromolecular crystallography can provide a beamsize that would have been considered small only a few years ago.

Reductions in beamsize place greater demands and constraints on beamline instrumentation and infrastructure. As the beamsize decreases, goniometry must become more precise and accurate, the resolving power of sample visualization must improve, the beam position must be tracked, with feedback implemented if necessary, and thermal stability becomes increasingly important. Commercial and in-house developments in all of these areas have allowed microfocus beamlines to flourish over the last decade.

Advances in other areas have also benefited microcrystallography. Automated sample exchange means that large numbers of crystals can be mounted in a single visit to the synchrotron. The benefits of being able to target a large number of samples are twofold. Firstly the screening and evaluation of large numbers of crystals is not time-prohibitive and secondly the requirement for a large number of crystals to form a single dataset is no longer a barrier. The limitation on the amount of data that can be collected from a single crystal is primarily imposed by Radiation damage (Holton and Frankel 2010). Based on the expected rate of decay of crystals, and the desired outcome of the experiment (*i.e.* molecular replacement or experimental phasing), Holton and Frankel provide a means of estimating the minimum crystal size required to obtain a complete dataset or alternatively, given a particular crystal size, the number of crystals required for complete data.^1^

The extremely limited lifetime of microcrystals in an intense microbeam means that care must be taken to optimise data collection. The use of a strategy program to exploit the symmetry of the reciprocal lattice minimizes the total rotation range required to obtain a complete dataset (Dauter 1999). The use of a sacrificial crystal to determine the sample lifetime at the beamline being used can be extremely valuable (Krojer and von Delft 2011), and provide a good idea of whether it will be possible to collect even the minimal rotation range from a single crystal. For extremely small crystals it is often possible to collect only a few degrees of data and the optimal approach is to collect from a large number of crystals without pre-orientation as screening images may use a large fraction of the total lifetime in the beam.

The finite lifetime of crystals in the X-ray beam can be addressed through the use of many crystals or, in the case when crystals are larger than the X-ray beam, by introduction of new material from the crystal into the X-ray beam. A simple means of achieving this is through the intentional offset of the center of rotation so that new material is continually introduced as the sample is rotated (Moukhametzianov et al. 2008). Another approach available at many beamlines is the line or helical scan. In this case a start and end point are defined and the crystal traces a helical path between these during data collection.

The advent of fast readout large area detectors has also facilitated throughput. The move from collecting a succession of single shuttered images to shutterless data collection represents a paradigm shift in macromolecular crystallographic data collection, reducing timing demands on instrumentation and reducing the duration of a dataset to a few tens of seconds (at most). While a strategy of collecting data with an oscillation range per image of half the crystal mosaicity was shown to be optimal a number of years ago (Pflugrath 1999), time and computing constraints meant that oscillation angles of 1° or more were typically used for data collection both in-house and at the synchrotron. The absence of a time penalty associated with collecting more frames per degree with fast readout detectors means that fine-phi slicing is now considered routine at synchrotron beamlines equipped with a pixel array detector (PAD). In addition to fast-readout, the characteristics of PADs such as small point spread function, lack of readout noise and dead-time during exposure mean data collection should be optimized to reflect these (Mueller et al. 2011).

A central tenet of the experimental setup of a diffraction experiment is the reduction of background scatter or noise. It has been shown that signal to noise in diffraction images can be improved by matching the beam size to the crystal size, this is particularly important for small micron-sized crystals. The gains made by matching the beamsize to the crystal can be dramatic as illustrated by data collection with two beamsizes from 5 × 5 × 5 μm^3^ polyhedra crystals (Evans et al. 2011a). By reducing the beamsize from a mismatched 8 × 8 μm^2^ to 4.5 × 5 μm^2^ data could be collected to higher resolution while at the same time reducing the absorbed dose.

Holton and Frankel (2010) describe how the presence of background scatter is an underlying reason for the large gap between the theoretical minimum crystal size (or minimum number of crystals required for a dataset) and the size of microcrystals from which structures have been determined. The approach described above of matching the beamsize to the crystal is one of the most obvious means of reducing background scatter but both the sample environment and sample preparation should also be optimized.

The benefits of minimizing the volume of solvent surrounding the crystal are twofold. Firstly, reducing the volume of non-diffracting material the X-ray beam passes through reduces diffuse scatter and secondly crystal alignment is simplified. Refractive effects can mean the apparent position of the crystal changes as the sample is rotated. For microcrystals this offset can be more than the size of the crystal or beamsize, making approaches such as diffraction based centering necessary. These approaches are described in more detail below but the need for them, or the time required to execute them, can be minimized by reducing the volume of solvent surrounding the crystal.

## *In situ* Data Collection

The collection of data *in situ*, i.e. directly from the Crystallisation platform, can provide a valuable tool in membrane protein crystallography. Given the increased numbers of reagents involved in the formation of crystals from membrane proteins and the typically extended optimisation process, the opportunity to interrogate with a synchrotron X-ray beam without recourse to sample manipulation and cooling is very appealing. This data collection method can thus dramatically speed up the feedback loop between initial crystal hits and ultimate structure determination. *In situ* data collection from SBS (Society for Biomolecular Sciences) standard footprint plates has been developed at a number of Macromolecular Crystallography (MX) beamlines at different synchrotron sources including the Swiss Light Source (Bingel-Erlenmeyer et al. 2011), European Synchrotron Radiation Facility (le Maire et al. 2011), Diamond Light Source (Axford et al. 2012) and Spring-8 (Kunio et al. 2013).

It has become clear that the technique is best used in conjunction with a microfocused X ray beam, since early crystal hits are typically small or poorly formed (Axford et al. 2012). Additionally, for the best information to be obtained, the crystallization platform needs to be designed with *in situ* data collection in mind in order to avoid excessive background scatter and to aid sample location. Increased uptake of the method has encouraged commercial suppliers to develop plates specifically designed for *in situ* crystallography, including the Greiner CrystalQuickX and the Mitegen *in situ*-1. These plates are designed for vapour diffusion experiments. A primary aspect of these designs is the use of thin, flat, low-X-ray-scatter materials to maximise the chance of measuring weak and short-lived diffraction at room temperature and to minimize the chance of optical aberrations that can lead to sample-beam misalignment. Crystallisation platforms designed around counter diffusion protocols can also be X ray compatible, with examples including CrystalSlide^TM^ (Ng et al. 2008) and CrystalHarp^TM^, the latter using quartz capillaries.

**Fig. 8.1:**
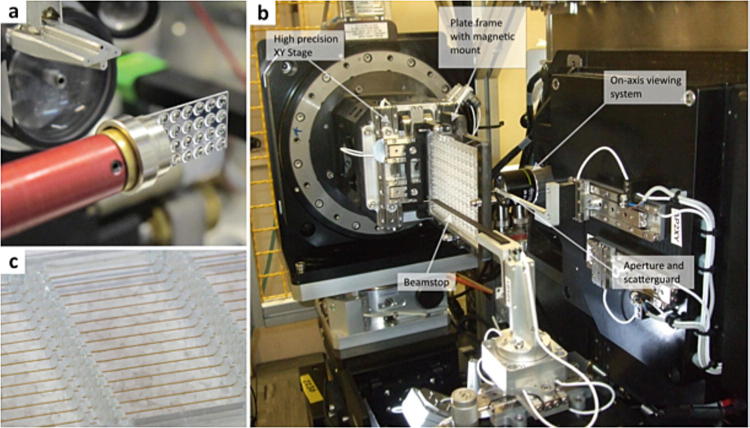
Example of platforms for *in situ* data collection. (**a**) X-CHIP (X-ray Crystallization High-throughput Integrated Platform) mounted on the GM/CA beamline at the Advanced Photon Source USA. (**b**). A Goniometer designed for SBS standard plates, with associated endstation components labeled, on beamline I24 at Diamond Light Source UK. (**c**) A close up of a CrystalHarp^TM^ plate displaying the array of detachable capillaries in which gradient diffusion crystallization can occur

Although the vast majority of Crystallisation plates are injection moulded from varieties of plastic, the most commonly used material for LCPLipidic mesophase crystallisation (LCP)Crystallisation platforms is glass, in a simple sandwich of two sheets. Glass is popular due to its stiffness, smoothness and low cost. However, 1 mm of glass transmits only 16 % of 12.4 keV X-rays, rendering it completely unsuitable for *in situ* experiments. The use of plastic films is becoming more common for the cover sheet since it does not shatter when cut for crystal fishing. If UV permissive plastic is also used as the support material UV-fluorescence detection of crystals is possible in addition to *in situ* data collection. Hybrid designs (Swissci AG) are also available in the form of multi-layer plates. In this case a lower glass support, that provides stiffness during handling, can be removed prior to *in-situ* data collection on the remaining thin sandwich of plastic containing the sample. Complementing the growth of dedicated facilities for *in situ* data collection from standard plates the development of smaller Crystallisation platforms allows *in situ* data collection on goniometry designed for conventional loops and pins. Examples of this include the X-CHIP (Kisselman et al. 2011) and modular plate designs, such as the CrystalSlide^TM^ and the CrystalHarp^TM^, where sections can be removed from a SBS format plate before mounting at the beamline, see Fig. 8.1.

In addition to sample screening, *in situ* data collection has the potential to be used for structure determination. Most recently, this has been demonstrated with the TecA integral membrane protein for which a structure was available from a conventional single crystal data set at 100 K. *In situ* crystallography enabled the determination of an equivalent room temperature structure via the merging of small wedges of data collected from 57 separate crystals but without the need for any sample manipulation (Axford et al. 2015).

## Crystal Alignment and Identification

The accurate alignment of crystals in the X-ray beam is a critical step prior to data collection. This can be particularly challenging on microfocus beamlines where the crystal and beamsize are much less than the size of a standard loop and these are both comparable to the visual resolution limit of the viewing system. Lensing effects and refraction at the surface and poor optical properties of mother liquor surrounding crystals can also combine to make crystal identification and alignment difficult. Alignment errors of only a few microns are sufficient to ensure crystals move out of the X-ray beam while being rotated and thus a number of approaches have been developed to ease crystal alignment. Image recognition can be used to speed up alignment, or automatically identify crystals (Karain et al. 2002; Lavault et al. 2006; Pothineni et al. 2006); however these rely on edge detection or contrast to identify the position of crystals (Bern et al. 2004), which can be hindered for example by the presence of precipitate (Cumbaa et al. 2003). It is particularly difficult to apply these routines to microcrystals however, when crystals may only be few pixels in size on on-line viewing systems, lipidic cubic phase (LCP)Lipidic mesophase crystallisation (LCP) provides an additional challenge. LCP provides a membrane-like environment for proteins (Landau and Rosenbusch 1996), and has proved to be a crucial tool in the Crystallisation of membrane proteins. A drawback of Lipidic mesophase crystallisation (LCP) is that upon cryo-cooling to 100 K it becomes opaque making the identification and centering of crystals in loops difficult (Cherezov et al. 2009). The popularity of Lipidic mesophase crystallisation (LCP) and limitations of visual alignment have led to the development of a number of tools for crystal identification and alignment at microfocus beamlines. Current methods for the alignment of microcrystals, and some future directions are outlined below.

### Current Methods for Crystal Alignment

#### Grid Scan

One of the most widely used methods of centring small or optically invisible samples is to use the diffraction grid scan (Cherezov et al. 2009; Song et al. 2007; Aishima et al. 2010; Bowler et al. 2010; Hilgart et al. 2011). This can be used to align small micro-crystals, crystals hidden in opaque Lipidic mesophase crystallisation (LCP) or to identify the best diffracting regions of larger crystals. The principle of this technique is to use a low dose X-ray beam to raster over the loop area. Diffraction images tagged with a position are then scored to indicate the best diffracting region(s). Diffraction images can be processed on the fly, and scored by, for example, the number of Bragg candidates present on the image using the spot finding software DISTL (Zhang et al. 2006). The scoring criteria look only at low resolution spots to avoid any ice rings present, and to help particularly with weakly diffracting samples. There are also options available to use a fluorescence raster scan, exploiting the presence of fluorescing elements present in the crystals (i.e. selenomethionine), with orders of magnitude lower intensity when compared to the diffraction grid scan. There are optimal strategies for exploiting the grid scan whilst minimizing the number of images collected and time required. The loop can first be scanned face on to determine the location of the crystal, with the crystal being centered to the best location. The loop can then be rotated through 90° with another scan being collected to determine the crystal location edge on. After being centered on both these positions the crystal is then centered and ready for data collection. It is vital that the lowest dose possible is used for these scans to ensure as little Radiation damage as possible prior to the data collection. This approach has proven very successful for centring membrane protein crystals on a number of different beamlines. Other strategies for grid scan alignment of crystals are explained further in the examples below.

#### UV Imaging

Ultraviolet (UV) fluorescence has routinely been used as a non-invasive method for distinguishing protein crystals in well plates. In the UV range from 260 to 320 nm, tryptophan, tyrosine and phenylalanine absorb the light, and native fluorescence is detected from approximately 300–450 nm (Lakowicz 2013). It is possible to distinguish between salt and protein crystals, with the protein crystals fluorescing brightly to give high contrast against the background (Judge et al. 2005; Calero et al. 2014). Advances in the technique have allowed the technique to be exploited at beamlines at the ESRF (France) (Vernede et al. 2006) and the Photon Factory (Japan) (Chavas et al. 2011) In the latter case they used pulses of UV light to ensure minimal damage to the crystals.

### Future Directions

#### X-ray Imaging

A technique that is being developed that works particularly well for membrane proteins is that of X-ray imaging. It is based on a well-studied technique where the absorption contrast of a material can be investigated using X-rays, but which isn’t currently routinely being used for macromolecular crystallography. Brockhauser *et al.* showed that it was possible to collect X-ray Microtomography data on soluble protein crystals on both high energy and MX beamlines at the ESRF (France). From this data they were able to reconstruct a three dimensional model of their crystal, surrounding liquor and loop to help with the addition of analytical absorption corrections (Brockhauser et al. 2008).

**Fig. 8.2:**
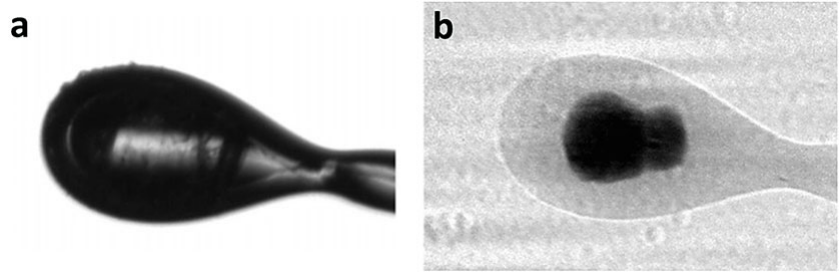
View of membrane protein crystals, the human A_2A_ adenosine G-protein coupled receptor in lipidic cubic phase mounted on a nylon loop, (**a**) using a visible microscope and (**b**) the same orientation of sample viewed as a radiograph. It is unclear from image (**a**) where the crystals are located, while after X-ray imaging (**b**) the presence and location of two crystals held within the loop becomes evident (Warren et al. 2013)

Warren et al. progressed this work by carrying out similar experiments on MX beamlines at the Diamond Light Source (UK) with membrane proteins in Lipidic mesophase crystallisation (LCP) to show how it was possible to image these loops and clearly distinguish where the crystals were mounted in the loop (Fig. 8.2). The paper described how this method was a lower dose method to the currently used grid scan and gives more information about the crystal size and shape to help determine how the beamsize should be altered for the subsequent data collection (Warren et al. 2013). Information on the three dimensional shape of crystals could also be used in the data scaling step, and this will prove to be particularly valuable for long wavelength data collection.

#### Non-linear Optical Imaging

**Fig. 8.3:**
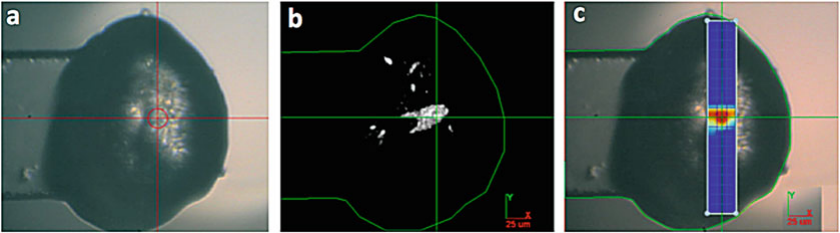
View of GPCRsG protein coupled receptors (GPCRs) membrane protein crystal in lipidic cubic phase. (**a**) Using a visible microscope. (**b**) The same orientation of sample viewed using SONICC and (**c**) the same sample located using grid scanning (Images provided courtesy of Garth Simpson, Purdue University)

Second-order nonlinear optical imaging of chiral crystals (SONICC)Second-order nonlinear optical imaging of chiral crystals (SONICC)SONICC has also recently been explored for imaging of membrane proteins in cubic phase. It works on the principles of second harmonic generation (Second harmonic generation (SHG)) in which the frequency of the light used is doubled. Constructive interference is observed from ordered chiral molecules, such as protein crystals, whereas crystals such as salt, or amorphous Lipidic mesophase crystallisation (LCP) will not produce a signal from the Second harmonic generation (SHG) (Kissick et al. 2010; DeWalt et al. 2013; Closser et al. 2013). SONICC has recently been installed on a beamline at the APS (USA) to image the crystals when mounted within loops. Here the method was compared to bright field images, and the current grid scan method to confirm it was possible to locate numerous membrane protein crystals within Lipidic mesophase crystallisation (LCP) (Madden et al. 2013), a visual comparison of the methods is shown in Fig. 8.3. The effects of Radiation damage were also considered due to the Lasers exposure on the crystals, however no significant effect was observed (Kissick et al. 2013). For more details regarding this method please see Chap. 10.1007/978-3-319-35072-1_7 of this book.

## Examples of Exploitation of Microbeams for Membrane Protein Structure Determination

The sections above briefly summarise some recent advances in synchrotron instrumentation and experimental approaches for making the most of microbeams. Below we describe some recent membrane structure determinations exploiting different aspects of these developments and highlighting best practice. Due to our affiliation these focus on work carried out at I24, Diamond Light Source as we can provide additional information on the experiments not necessarily found in the materials and methods sections. The approaches used and described are however broadly applicable to membrane protein structure determination at microfocus beamlines worldwide.

### The Class B G protein coupled receptors (GPCRs) Corticotropin – Releasing Factor Receptor 1

The structure of the class B human G protein coupled receptors (GPCRs) corticotropin – releasing factor receptor 1 (CRF_1_R) was determined at I24, Diamond Light Source. The structure and function of this G protein coupled receptors (GPCRs) is described by (Hollenstein et al. 2013); we here highlight some of the experimental approaches exploited to allow data to be collected to a resolution that allowed identification of an unexpected binding pocket.

**Fig. 8.4:**
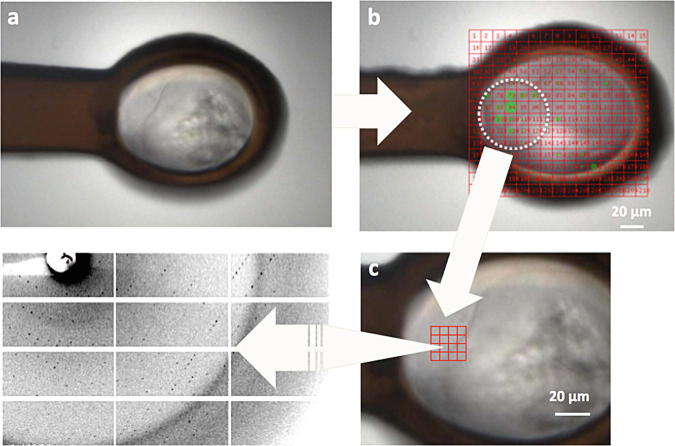
Strategy for locating crystals in opaque. After the crystal is mounted (**a**), a grid with a pitch of 10 μm is drawn that covers the entire loop (**b**). Following on-the-fly scoring using DISTL promising sub-areas are identified. In the case of small crystals, a second grid can be drawn (**c**) and, with a concomitant decrease in the beamsize, the position of the crystal is more accurately determined. In the example shown here the pitch of the second grid is 5 μm. The resolution at the edge of the diffraction image shown is 3 Å

Crystals of CRF_1_R were grown in Lipidic mesophase crystallisation (LCP) with a maximum dimension of approximately 15 μm. Upon Cryocooling the Lipidic mesophase crystallisation (LCP) became opaque and in order to align crystals to the X-ray beam the diffraction grid scan was used (Fig. 8.4). A single grid scan was typically sufficient to align larger crystals (approx. 10 microns in size or larger), but for small crystals (~5 μm) an iterative approach was required. Following the initial gridscan, the beamsize was reduced to ~5 × 5 μm^2^ to better match the crystal size and a second grid scan carried out around any hits found in the first gridscan. For all gridscans the X-ray beam was attenuated to minimise Radiation damage to crystals. Once centered in the X-ray beam CRF_1_R crystals diffracted to ~3 Å, but only with long exposure times (7.5 s per degree of oscillation). This corresponds to an absorbed dose of ~ 6 MGy per second. Radiation damage was therefore a limiting factor and it was only possible to collect a small wedge (2–3°) of data from each crystal. A complete dataset was built up using the microdiffraction assembly method (Hanson et al. 2012). In this approach data from each crystal are split into 1° bins. These bins are then iteratively scaled and merged to a reference low resolution (4.3 Å) dataset using XSCALE. Using this approach a complete dataset to 2.97 Å was built up containing data from 30 crystals. This compares with a 3.15 Å dataset formed using a conventional assembly method.

### Structures of ABCB10, a Human ATP-Binding Cassette Transporter in Apo- and Nucleotide-Bound States

The structure of ABCB10, one of three mitochondrial ATP-binding cassette (ABC) transporters has been determined using microfocus crystallography. These are located in the inner membrane of mitochondria and were found to adopt an unexpected conformation when complexed with nucleotide analogues, compared to previously reported structures. Further details about the differences in conformation, as well as a more detailed description of the structures are described by (Shintre et al. 2013). All structural data were collected on I24 at Diamond Light Source. Initial structure solution was achieved using plate-like crystals which were phased by isomorphous replacement with a single mercury derivative (SIRAS). These crystals were highly anisotropic. Data isomorphism and diffraction quality was significantly improved by flash cooling crystals at 6 °C instead of their growth temperature of 20 °C, which led to reproducible diffraction to around 3.4 Å in the best direction and 4.4 Å in the worst. Over 500 crystals were screened to optimize the Crystallisation and freezing conditions, followed by a further 300 crystals to find a suitable heavy atom derivative. Due to the anisotropy and disorder of the nucleotide binding domain, the model building could not be completed. A different rod-shaped crystal form was obtained with protein purified in the presence of lipid which diffracted isotropically to 2.9 Å. It was possible to phase these crystals using molecular replacement, using the plate form structure as a model and complete the structure.

For the plate-like crystals, all data collections utilised the helical line scan on I24. During this form of data collection the crystal is translated whilst it is rotating so that the entire length of the crystal is exploited and new material is continually introduced into the beam. This technique minimises crystal degradation and it may even be possible to outrun Radiation damage. For sample alignment both ends of the crystal need to be well centred defining a path along the crystal for the data collection.

**Fig. 8.5:**
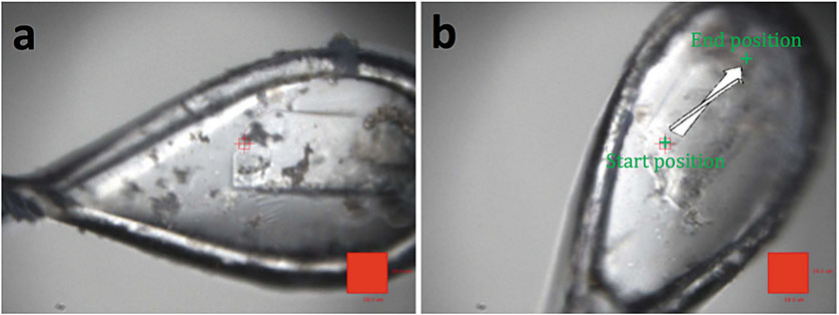
Plate-like crystal of ABCB10 before (**a**) and after (**b**) the loop was bent for data collection. (**b**) Shows the centered start and end positions for the line scan data collection

Due to a relatively long unit cell *c* axis, which coincided with the thinnest dimension of the plate, the mounting loop needed to be bent manually with a pipette tip to align the *c* axis closer to the rotation axis to minimise overlaps (Fig. 8.5). The start and end points of the helical scan were determined by two perpendicular line scans at each end of the crystal. Visual alignment was not possible due to the loop reorientation and limited thickness of the plates. In the majority of cases, the beam was shaped to a vertical letter box (30–50 μm high × 10 μm wide) to illuminate a larger portion of the crystal.

For the rod-shaped crystal form, broad grid scanning was used to identify the best diffracting regions of each crystal. In every case, the best diffraction was obtained from either end of the rods (dimensions 20 × 20 × 150 μm^3^) with much weaker diffraction from the central portion. Data to 2.85 Å were collected using a 10 × 10 μm^2^ beam and rotating about a single portion of crystal.

### Structure of the Integral Membrane Protein Diacylglycerol Kinase (DgkA)

DgkA is functionally unique enzyme catalysing the conversion of diacylglycerol and ATP to phosphatidic acid and ADP and enabling membrane phosolipid turnover (Li et al. 2013). After initial trials crystals were produced in lipidic cubic phase at 4 °C for wild type, a thermally stable mutant and a seleno-methionine (SeMet) thermally stable mutant. Maximum crystal dimensions were 50 × 50 × 50 μm^3^ bipyramids for the wildtype, 50 × 75 × 100 μm^3^ bypyramids for the thermally stable mutant and 10 × 50 × 150 μm^3^ rectangles for the SeMet derivative. Crystals were harvested with 30–100 μm micromounts (Mitegen) in a 4 °C cold room and flash-cooled in liquid nitrogen. Data were collected at the 23-ID-B GM/CA-CAT beamline at the Advanced Photon Source, Argonne, and also at beamline I24 at Diamond Light Source. On both beamlines a 10 × 10 μm^2^ beam was used, though this was realised by different means at each beamline. At GM/CA-CAT a collimator was used (Fischetti et al. 2009), while at I24 a two-stage demagnification was used. In both cases, raster scanning with a tenfold attenuated beam was used to locate and identify the most ordered regions of a crystal. The highest resolution dataset was obtained by combining a complete low-resolution dataset (3 Å) from a single crystal with 18 wedges of 10° of high-resolution and 10° data from Multiple crystals.

Xia2 was used to integrate diffraction data and the best wedges were identified by R-value and isomorphic cell parameters. Anomalous data were obtained in a similar way by merging 19 separate data collections from SeMet crystals to build up a 200 fold redundant dataset to 3.0 Å. Seventeen putative Se sites were identified using SHELX C/D/E (Sheldrick 2008) and phases extended using PHENIX (Adams et al. 2002) to 2.05 Å, representing the highest resolution native data.

A previously published solution phase NMRNuclear magnetic resonance (NMR) structure failed as a model for molecular replacement and was seen to be significantly different to the final crystal structure. Subsequent solid-state Nuclear magnetic resonance (NMR) spectroscopy studies within Lipid bilayer have supported this crystallographic structure, highlighting the importance of working in effective membrane mimetic environments such as Lipidic mesophase crystallisation (LCP) (Murray et al. 2014).

### Structural Basis for Outer Membrane Lipopolysaccharide Insertion

Beta barrel membrane proteins often prove difficult targets for molecular replacement due to the wide variation in strand number and propensity for motion in the barrel. This necessitates an experimental phasing approach with the initial map quality of key importance in ensuring successful main chain tracing.

The LptD/E complex performs the final stage in insertion of the mature lipopolysaccharide (LPS) into the outer leaflet of the outer membrane in Gram-negative bacteria (Dong et al. 2014). LptE sits inside the 26 β-strand LptD barrel acting as both a plug for the barrel and as guide for correct LPS orientation. SeMet labelled crystals (110 × 110 × 170 μm^3^) of the LptD/E complex (87.2 kDa, 20 selenium atoms) were obtained in space group *I*2 with unit cell *a* = 173.4 Å, *b* = 76.1 Å, *c* = 213.6 Å and *β* = 111.5 °. The solvent content was approximately 67 % with two complexes per asu. The X-ray diffraction from these crystals was anisotropic with CC_1/2_ > 0.5 cutoffs of h,l = 2.80 Å and k = 3.9 Å.

**Fig. 8.6:**
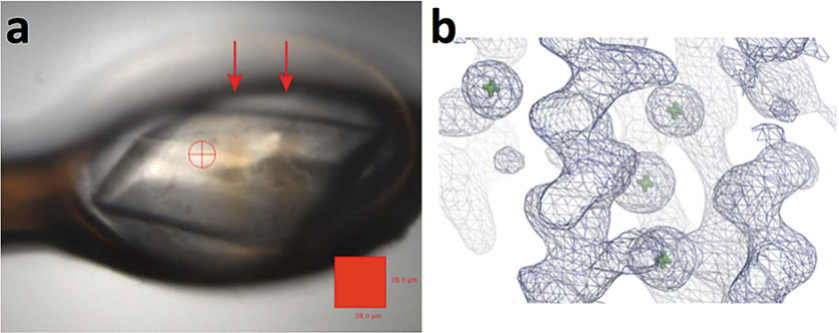
(**a**) LptD/E crystal after two data collection slices. The exposed areas are indicated with *red arrows* and the 20 × 20 μm^2^ beam by the *red circle* and *crosshairs*. (**b**) Section of the LptD/E density modified map contoured at 0.09 e Å^−3^ (1.5 rmsd) showing part of the electron density for the LptE helix and strands. Selenium sites are indicated by a *green cross*

The strategy used for structure solution was to collect multi-wavelength anomalous diffraction (MAD) data to provide a high quality initial map but the low symmetry of the *I*2 space group and weak diffraction meant that high anomalous multiplicity would be difficult to achieve without significant Radiation damage on a single crystal. The approach chosen was to use the microfocus capabilities of beamline I24 at Diamond Light Source to divide the crystal into four regions with a 20 × 20 μm^2^ beam and collect 360° of fine-sliced data (0.1° oscillation) at one of the four MAD wavelengths (peak, inflection, high remote and low remote) in each region (Fig. 8.6a). To achieve the best possible experimental map, this approach was repeated on three isomorphous crystals resulting in the collection of around 45,000 frames in total. All data were processed using XDS (Kabsch 2010) to 2.8 Å resolution and the individual wavelengths combined to give ~16-fold anomalous multiplicity. All 40 selenium sites were identified using SHELXC/D through autoSHARP (Vonrhein and Schulz 2007). Subsequent density modification with DM gave a starting map with CC = 0.68 (Fig. 8.6b) into which all of LptE and most of the pore strand main chain from LptD could be automatically built with Buccaneer (Cowton 2006).

## Concluding Remarks

The challenges of membrane protein crystallography often continue beyond the well-documented difficulties of Crystallisation, with weak and often anisotropic X-ray diffraction and low overall resolution hindering structure solution. Difficulties with Crystallisation can be partly addressed through the use of *in situ* data collection which removes confounding factors such as mechanical stress during loop mounting and damage during the Cryocooling process when assessing the promise of initial crystal hits. A microfocussed beam allows this characterization to be done at an early stage of the crystal optimisation process.

The challenges associated with collecting high quality diffraction data from small or badly ordered crystals can be partially addressed through the use of a microbeam. Microbeams themselves give rise to challenges not least because of beam induced damage and the difficulties associated with sample alignment. These challenges can be addressed in part through the use of many crystals to form a single complete dataset and exploitation of on-line tools such as the grid scan or tomography for alignment.

The use of beamsizes of less than 10 microns is now the norm for many membrane protein crystallographers. Further developments in synchrotron sources and beamline instrumentation will see both a more widespread availability of microfocus beams worldwide and also the provision of even smaller, more intense microbeams. (Many) multi-crystal strategies for structure solution will become standard practice and the ever-decreasing lifetime of crystals in the beam will mean new modes of sample delivery such as liquid jets or fixed aperture arrays are ported from sources such as the free electron Lasers for use at synchrotrons. Taken as a whole, advances in instrumentation and methods will help ease the pathway to structure solution for ever more challenging targets in membrane protein crystallography at synchrotron sources.

## Footnotes

1 Online calculator available at http://bl831.als.lbl.gov/xtalsize.html.
